# Quantitative interpretation of bone marrow biopsies in MPN—What's the point in a molecular age?

**DOI:** 10.1111/bjh.19154

**Published:** 2023-10-19

**Authors:** Hosuk Ryou, Oliver Lomas, Helen Theissen, Emily Thomas, Jens Rittscher, Daniel Royston

**Affiliations:** ^1^ Nuffield Division of Clinical Laboratory Sciences, Radcliffe Department of Medicine University of Oxford Oxford UK; ^2^ Department of Haematology Oxford University Hospitals NHS Foundation Trust Oxford UK; ^3^ Department of Engineering Science, Institute of Biomedical Engineering (IBME) University of Oxford Oxford UK; ^4^ Ground Truth Labs Oxford UK; ^5^ Oxford NIHR Biomedical Research Centre Oxford University Hospitals NHS Foundation Trust Oxford UK; ^6^ Ludwig Institute for Cancer Research University of Oxford Oxford UK; ^7^ Department of Pathology Oxford University Hospitals NHS Foundation Trust Oxford UK

**Keywords:** artificial intelligence, bone marrow biopsy, clinical trials, marrow fibrosis, megakaryocytes, myeloproliferative neoplasm

## Abstract

The diagnosis of myeloproliferative neoplasms (MPN) requires the integration of clinical, morphological, genetic and immunophenotypic findings. Recently, there has been a transformation in our understanding of the cellular and molecular mechanisms underlying disease initiation and progression in MPN. This has been accompanied by the widespread application of high‐resolution quantitative molecular techniques. By contrast, microscopic interpretation of bone marrow biopsies by haematologists/haematopathologists remains subjective and qualitative. However, advances in tissue image analysis and artificial intelligence (AI) promise to transform haematopathology. Pioneering studies in bone marrow image analysis offer to refine our understanding of the boundaries between reactive samples and MPN subtypes and better capture the morphological correlates of high‐risk disease. They also demonstrate potential to improve the evaluation of current and novel therapeutics for MPN and other blood cancers. With increased therapeutic targeting of diverse molecular, cellular and extra‐cellular components of the marrow, these approaches can address the unmet need for improved objective and quantitative measures of disease modification in the context of clinical trials. This review focuses on the state‐of‐the‐art in image analysis/AI of bone marrow tissue, with an emphasis on its potential to complement and inform future clinical studies and research in MPN.

## Graphical abstract

Quantitative analysis of bone marrow tissue features in myeloproliferative neoplasms (MPN) enables cellular and stromal components to be objectively assessed and compared across patient cohorts. This improved morphological feature analysis can inform and refine our understanding of the microenvironment in the marrow stem cell niche(s). Combining whole sample analysis with detailed cell–cell and cell–stromal interactions improves our disease modelling of MPN and provides a rational approach for improved diagnosis and disease classification. In turn, accurate and objective morphological correlates of therapeutic response can be developed that enable clinicians to monitor the response of individual patients in the context of large sample cohorts (cohort indexing).

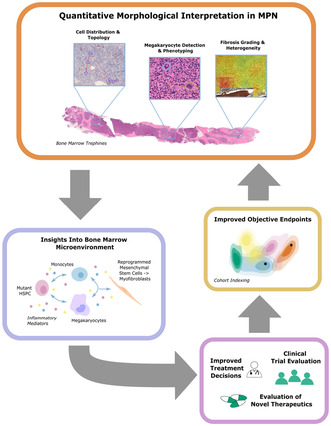

## INTRODUCTION

The classical Philadelphia chromosome‐negative (Ph^−^) myeloproliferative neoplasms (MPN) are characterised by the overproduction of blood cells derived from haematopoietic stem and progenitor cells (HSPCs) that harbour mutations resulting in cytokine‐independent or hypersensitive proliferative signals. The three most common MPNs are essential thrombocythaemia (ET), polycythaemia vera (PV) and primary myelofibrosis (PMF), each with overlapping clinical and laboratory findings that can make their distinction challenging, particularly at early disease time points.^1^, ^2^, ^3^ In over 90% of MPN, a driver mutation in the genes encoding *JAK2*, *CALR* or *MPL* results in constitutive activation of the *MPL‐JAK–STAT* signalling pathway.^4^, ^5^, ^6^ Despite this commonality, the disease phenotype and risk of progression in MPN are highly variable.^7^, ^8^ This is partly determined by enhanced mutational allele frequencies of oncogenic drivers or the co‐occurrence/acquisition of additional mutations within the clonal population. Genes encoding transcriptional regulators, epigenetic regulators, splicing factors and apoptotic signalling pathways are all implicated in disease progression in MPN and ultimately contribute to the disease phenotype.^9^, ^10^ However, it is increasingly recognised that intimate and complex cellular and stromal interactions within the bone marrow microenvironment are also central to the very earliest stages of MPN initiation.^11^, ^12^, ^13^, ^14^ Indeed, inflammatory changes occurring within the stem cell niche, fuelled by monocytes and megakaryocytes, may even precede the acquisition of driver mutations in MPN.^15^, ^16^ In the context of primary myelofibrosis (PMF), the differentiation of mesenchymal stem cells (MSC) to fibrosis‐driving myofibroblasts appears to be dependent upon these initial inflammatory changes.^17^, ^18^, ^19^ Subsequent progression to severe marrow fibrosis determines the distinctive clinical features of PMF, with hallmark cytopenias, extra‐medullary haematopoiesis, hepatosplenomegaly and cytokine‐driven systemic symptoms.^20^, ^21^

Recent insights into the HSPC‐intrinsic factors and their interactions with the marrow microenvironment have driven the development and evaluation of numerous novel therapeutics in MPN.^22^, ^23^ It is anticipated that such agents and/or novel combinations will better target the underlying cell–cell and cell‐stromal pathological processes responsible for refractory disease, therapeutic resistance and disease progression that are frequently encountered in MPN. Notwithstanding these advances, questions remain over the adequacy of existing strategies for evaluating new therapies and defining therapeutic response. In particular, attention has been drawn to the absence of consistent and standardised parameters by which disease modification is measured in MPN.^24^ Amongst other parameters, the need for accurate assessment of bone marrow trephine (BMT) features using robust and reproducible measures of tissue response have been highlighted. Without such, there is a serious risk of incorrectly evaluating emerging clinical trial data. This may lead to the early abandonment of viable therapeutics and/or the promotion of agents offering little or no benefit to patients.

The remainder of this article will focus on the potential for improved bone marrow biopsy analysis in MPN. It will outline the opportunities to improve the detection and quantitation of tissue features relevant to diagnosis and classification in clinical practice and the evaluation of disease modification in the context of clinical trials. Particular consideration will be given to the emerging application of image analysis techniques powered by machine learning (ML) and artificial intelligence (AI).

## EVALUATION OF BONE MARROW BIOPSIES IN MPN—WHERE ARE WE NOW?

Despite advances in our understanding of the pathobiology of MPN, accurate diagnosis and assessment in routine practice remain dependent upon an integrated approach incorporating clinical, morphological, immunophenotypic and genetic findings.^2^, ^3^ The conventional microscopic assessment of BMTs remains crucial as it provides unparalleled information on marrow cellularity, the topology of haematopoietic cell lineages and their maturation, the marrow stroma and bony structures. Much of this information is obtained with standard haematoxylin and eosin (H + E), Giemsa and silver‐stained BMT sections that are readily prepared in most diagnostic laboratories. These tinctorial stains, typically complemented by a panel of immunostains directed towards individual cell populations, remain the core of MPN tissue diagnosis. In stark contrast to the emergence of highly sensitive quantitative techniques for the detection of genetic aberrations in blood cancer, the morphological terminology used within the current classification schema for MPN remains subjective, qualitative and variably reproducible. This reflects the traditional practice of haematopathology, with haematologists/haematopathologists challenged to condense and assimilate highly complicated, multifaceted tissue features into a text‐based report. These reports typically include very limited semi‐quantitative data and follow a standardised format (varying between institutions) that employs fixed terminology drawn from an accepted lexicon of descriptive terms. Diagnostic manuals and companions to the WHO and ICC classification schemes typically include only selected images that correspond to these descriptive terms; single or clustered megakaryocytes with features corresponding to each of the ‘idealised’ MPN subtype descriptions are often accompanied by exemplar fibrosis grade (WHO MF) reticulin/collagen images.^25^, ^26^, ^27^, ^28^ However, experienced practitioners recognise that significant heterogeneity is seen within most MPN samples, with even ‘normal’/reactive BMT samples often defying these simplistic schemas.

Over the years, several studies have sought to evaluate the consistency of BMT feature assessment by haematopathologists. Some have demonstrated relatively poor interobserver concordance in the identification and interpretation of core feature assessments.^29^, ^30^, ^31^ In the context of fibrosis (a key component of MPN assessment), studies of concordance in WHO MF grade assessment have been shown to be highly dependent upon the level of expertise and extent of collaboration amongst participants.^32^, ^33^ Similarly, the documented high level of concordance in pre‐PMF diagnosis reported by several European studies appears to be strongly associated with the collaborative experience of the contributing centres.^34^, ^35^ Regardless, it is widely recognised that many haematologists and haematopathologists have difficulty in consistently applying the WHO morphological criteria for MPN. Notwithstanding these concerns, even the accurate application of formal diagnostic criteria only allows for the assignment of limited, discrete diagnostic entities and non‐linear grading categories. The spectrum of morphological features encountered within individual MPN patient biopsies simply cannot be captured and quantitated using current criteria.

## APPLYING AI TO BONE MARROW INTERPRETATION

### Overview

AI has found increasing application in various fields of medicine and can be broadly defined as the study and development of computer systems capable of performing complex tasks that ordinarily require human intelligence.^36^ With the widespread adoption of whole slide scanners, subfields of AI dedicated to various image analysis problems have been applied to pathology. These approaches typically employ algorithms capable of estimating certain bespoke morphological criteria from the data.^37^, ^38^, ^39^ Taken together, such activities are referred to as ML and often employ deep neural networks comprising multiple layers of artificial neurons that mimic human learning. By repeatedly updating the parameters of these artificial neurons, such neural networks can be trained to learn their own level of data representation in a process referred to as deep learning (DL).^40^, ^41^

Several experimental algorithms have been developed to automate the detection and cytomorphological description of distinct cell populations within peripheral blood and marrow aspirate smears in haematological disease.^42^, ^43^, ^44^, ^45^, ^46^, ^47^ The evaluation of BMT samples is significantly complicated by the variation in object shape due to microtome cutting and the presence of ‘higher order’ tissue architecture that is absent from liquid samples. However, several groups have successfully employed ML approaches to identify and quantitate specific cell populations within BMTs using immunostaining or routine H + E images.^48^, ^49^, ^50^, ^51^, ^52^ The segmentation of haematopoietic cells into myeloid, erythroid and megakaryocytic lineages has also been complemented by the detection of fat spaces and bone tissue using periodic acid‐Schiff‐stained slides as part of a more comprehensive description of normal marrow tissue.^53^ In the context of myeloid malignancies, ML has been applied to marrow tissue in myelodysplastic neoplasms (MDS) using tissue microarrays.^54^ Morphological features extracted from H + E images were used to predict relevant genetic abnormalities and demonstrated promise in the discrimination of MDS and MDS/MPN from reactive samples. The broad application of AI to bone marrow tissue analysis and the attendant challenges have recently been reviewed.^55^

### Using AI to improve conventional BMT interpretation in MPN

**FIGURE 1:**
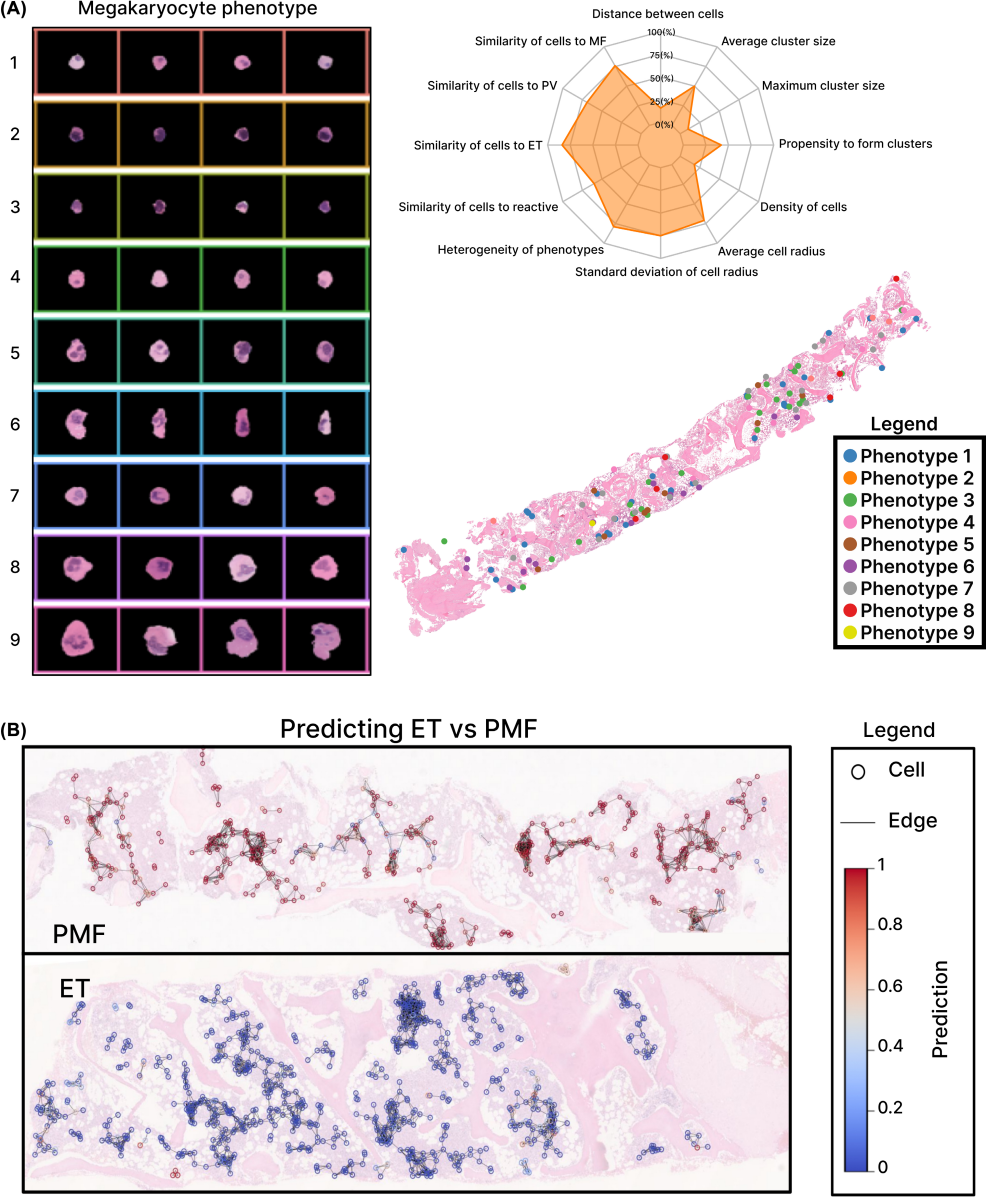
Representation of megakaryocytes within BMT samples. (A) Nine phenotypes were initially identified using unsupervised clustering analysis, with phenotypes 8 and 9 being readily identified as typical of the large megakaryocytes with polylobated nuclei frequently encountered in many MPNs. The megakaryocyte population within a sample can be graphically represented as a radar plot showing each of the analysed cell features or overlaid directly onto a BMT image (panels adapted from ref. [56] with permission from *Blood Advances*). (B) Overlays of the predictions from the graph‐based neighbourhood model on exemplar essential thrombocythaemia (ET) and primary myelofibrosis (PMF) samples. The megakaryocytes are represented by circles, with the colour denoting the prediction outcome of the binary classification between ET and PMF (applicable to any pairwise comparison of MPN or reactive samples). The edges of the constructed graphs, representing the feature similarity of megakaryocytes within cell communities, are indicated by lines between cells.

We have recently employed DL approaches to improve the analysis of megakaryocyte features in BMTs, aiming to distinguish ‘normal’/reactive appearances from those encountered in MPN, and to distinguish between important MPN subtypes.^56^ Using an initial subset of manually annotated images, DL methods were applied to detect and delineate megakaryocytes, resulting in a curated library of >60 000 cells. Clustering analysis was performed on this library to identify nine candidate cell phenotypes. Although these phenotypes were derived in a data‐driven fashion using DL, several phenotypes displayed features readily recognisable as typical of either a reactive marrow or MPN. The phenotypic and topographical profiles of these megakaryocytes were extracted and used to create abstract representations of each patient trephine sample (Figure 1A). A random forest classifier was subsequently trained on these representations to distinguish between MPN and reactive samples (area under the curve [AUC] = 0.98). This work confirmed a clear association between megakaryocyte cytomorphology/topology and the MPN disease subtype and provided evidence of important mutational–morphological associations. Subsequent work employed a multiscale graphical representation to provide context to local collections or communities of megakaryocytes (Figure 1B). This highlighted the importance of the local tissue microenvironment in the interpretation of individual cellular features. By integrating disease‐relevant megakaryocyte morphology with overall tissue topology using a graph neural network, MPN disease subtypes could be identified purely on the basis of the megakaryocyte population (accuracy[PV/ET] = 0.94; accuracy[ET/PMF] = 0.93; accuracy [PV/PMF] = 0.90).^57^, ^58^ By applying graph metrics to the detected cell communities, we obtained a quantitative description of the local context of the megakaryocyte population. Importantly, visualisation of these megakaryocyte communities within the context of the tissue biopsy is highly intuitive and well suited to integration with conventional BMT assessment.

**FIGURE 2:**
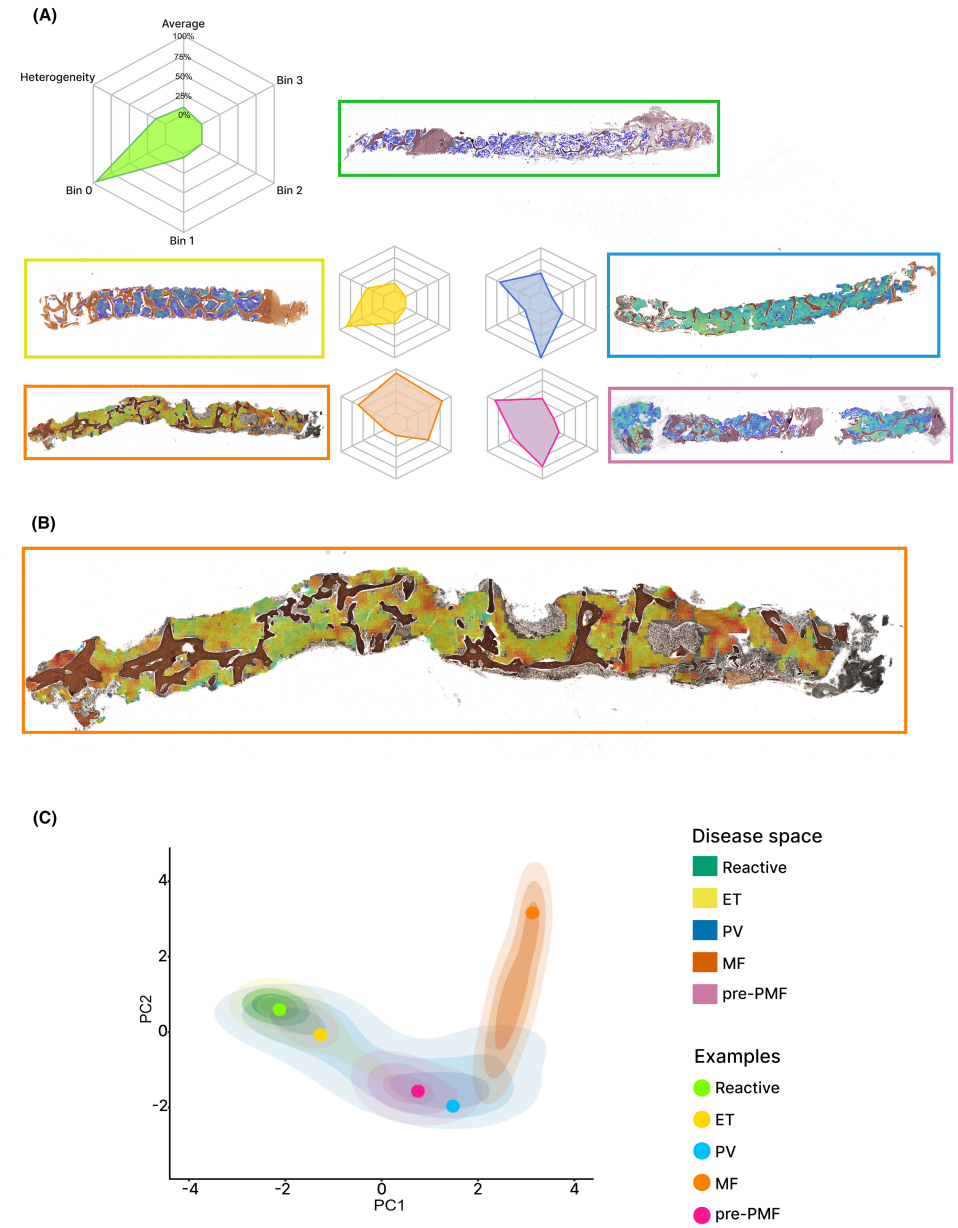
Representation of fibrosis within BMT samples. (A) Examples of false‐coloured fibrosis heatmaps overlaid onto original reticulin‐stained BMT images. Radar plots capture the average continuous indexing of fibrosis (CIF) tile score, tile distribution across four bins (broadly corresponding to WHO MF grades 0, 1, 2 and 3) and heterogeneity of tile distribution. (B) Enlarged example of a fibrosis heatmap demonstrates the severity and heterogeneity of fibrosis frequently encountered in primary myelofibrosis (PMF). (C) Principal component analysis (PCA) plot of the abstract representations of sample fibrosis reveals clustering of reactive cases and MPN subtypes. The features captured in PC1 and PC2 are the average sample tile CIF score, tile distribution and heterogeneity of tile distribution. Individual patient samples (coloured circles) are plotted against the PCA disease space.

Following automated megakaryocyte detection and analysis, we next sought to refine the assessment of marrow fibrosis in MPN.^59^ Briefly, we extracted uniformly sized tiles from images of reticulin‐stained BMT sections that were then ordered using a ranking DL model. The output scores of this ranking model, corresponding to the severity of fibrosis, were then mapped onto BMT images. We refer to this approach as ‘Continuous Indexing of Fibrosis (CIF)’, and it allows the generation of a CIF map where fibrosis within BMT samples can be intuitively visualised as a heatmap showing both the severity and heterogeneity of fibrosis (Figure 2A,B). This strategy enabled us to identify microfoci or ‘hotspots’ of advanced fibrosis that could discriminate between important MPN subtypes (including ET and pre‐PMF) and aid in the identification of patients at risk of fibrotic progression (AUC = 0.77). Importantly, these features were not discernible by experienced haematopathologists using conventional BMT assessment and are not captured in current fibrosis grading criteria. The visualisation output of the fibrosis analysis is readily combined with conventional microscopic assessment and can even be integrated with the megakaryocyte features to create a single multifeature output.

Of note, the training and validation phases of AI development for this work only utilised samples deemed diagnostically adequate for assessment by specialist haematopathologists. Determining sample adequacy in BMT is subjective and varies between haematopathologists, with some applying arbitrary minimum requirements of intact intertrabecular spaces or biopsy length. The potential role and utility of AI‐based approaches in such evaluations remain unclear. Samples whose staining significantly impaired interpretation were re‐stained until adequate for conventional assessment. Similarly, samples in which there was insufficient marrow sampling or tissue artefacts (crush, folding, tearing, etc.) precluded conventional assessment were excluded from our AI training and validation sets. Minor foci of such artefacts within otherwise interpretable samples were manually annotated and excluded from analysis, although such features are well suited for future AI‐based detection and automated exclusion.

### Learning to ‘see’ the marrow in MPN—Democratising tissue diagnosis?

In most studies applying AI technologies to biopsy interpretation there has been a focus on replicating or improving the description and classification of patient samples in line with conventional microscopic assessment. This approach allows for the application of existing diagnostic criteria and integration into current reporting guidelines; it is generally welcomed by clinicians and pathologists as familiar and readily comprehensible. However, AI‐based strategies have the potential to identify, quantitate and represent features that transcend this approach. In the context of MPN, this potential is evident when one considers the challenge of accurately identifying morphological progression across serial BMT samples. This is commonly encountered in patients for whom repeated biopsies are taken to specifically monitor disease or establish evidence of treatment response. Using the extracted megakaryocyte and fibrotic features outlined above, we demonstrated the potential of representing individual patient samples in the context of large patient cohorts that span the morphological spectrum of MPN.^56^, ^59^, ^60^ This process employs an intuitive two‐dimensional visualisation of so‐called ‘disease space’ using principal component analysis (PCA). This disease space provides an informative reference for individual patient samples (Figure 2C). By directly comparing patient samples to the reference cohort (‘Cohort Indexing’), classification categories can be quickly determined, along with inferences about the potential for disease progression or regression. This approach is particularly well demonstrated using sequential biopsy samples, in which feature progression can be ‘plotted’ against disease space. This visualisation of BMT samples is objective, quantitative and impervious to the intraobserver and interobserver variability encountered when haematopathologists attempt to compare features across samples. Importantly, cohort indexing can be used to interrogate single tissue features or combine multiple related features to enable integrated evaluation of a patient sample. This flexibility has application in both routine clinical assessment and clinical trial evaluation, where discrete changes are of relevance for the evaluation of specific drug mechanisms of action. These may include improvement in marrow fibrosis, normalisation of marrow cellularity and/or megakaryocyte maturation.

In addition to improving the assessment of single and sequential MPN samples, AI‐driven cohort indexing facilitates the interpretation of key biopsy features without the need for expertise in tissue microscopy. In principle, such approaches may ultimately enable non‐specialist healthcare professionals and patients to directly review BMT biopsy results and extract salient diagnostic and prognostic features, albeit with a specialist haematopathologist assuming ultimate responsibility for formal interpretation.

In considering the future clinical application of such diagnostic megakaryocyte and fibrosis algorithms, questions remain over how such algorithms might be deployed. In turn, care must be taken to ensure that any proposed validation exercises (+/− prospective trials) are appropriate and reflect the proposed clinical application. It is our contention that the purpose and utility of the AI approaches reviewed and summarised here is not to provide a ‘blind’ comprehensive analysis of all BMTs received by diagnostic departments. Rather, we consider that our AI‐based approaches are best employed when the key purpose of bone marrow evaluation is to confirm or refute an informed clinical suspicion of MPN. In this respect, AI‐based evaluation of MPNs should be viewed as an ancillary tool in the armoury of diagnostic haematopathologists and haematologists. We do not consider it a candidate replacement for specialist reporting or a general diagnostic screening tool for marrow assessment. In developing the algorithms outlined above, we therefore utilised training and validation data from BMT samples in which there was a specific putative or established diagnosis of MPN. Samples from patients with diagnoses capable of morphologically mimicking MPN (e.g. ITP treated with thrombopoietin receptor agonists, systemic inflammatory or autoimmune disorders and other malignancies) were deliberately excluded. However, we recognise that such conditions can co‐occur or complicate bone fide MPN and are frequently captured under the classification of MPN, not otherwise specified (MPN, NOS) or MPN, unclassifiable (MPN‐U).^2^, ^3^, ^61^ These challenging cases require exhaustive clinical and laboratory correlation and sometimes repeat biopsy after periods of observation in order to confirm a diagnosis of MPN. However, with the application of rigorous diagnostic criteria, they are thought to represent less than 5% of all MPNs,^62^ making them rare and challenging to curate and collect for the purposes of AI algorithm development. To date, we have therefore not included these entities in our work.

## LEVERAGING THE BMT BIOPSY—CLINICAL END‐POINTS IN MPN AND THE EVALUATION OF NOVEL THERAPEUTICS

### Disease progression and prognostication in MPN

The key aims of any therapeutic strategy in MPN are to ameliorate patient symptoms, minimise vascular events and delay/prevent disease progression.^63^ Clinical indicators of MPN progression include aggravation of perturbed blood counts, worsening constitutional symptoms and the occurrence or intensification of thrombohaemorrhagic events.^7^ An associated increase in the blast cell population (>20% blasts in the peripheral blood or bone marrow) indicates blast phase MPN (MPN‐BP). Such disease transformation resembles secondary AML but is often refractory to conventional AML therapies, with a dismal prognosis of under 6 months.^64^ Chronic MPNs also have the potential to progress to each other, with the development of post‐ET MF and post‐PV MF (termed secondary MF [sMF]) occurring in approximately 10%–15% of patients.^65^, ^66^ The progression of prefibrotic PMF (pre‐PMF) to PMF appears to be significantly higher, with a cumulative risk of 35%–40% after 15 years.^35^ The clinical importance of myelofibrotic progression in MPNs is underscored by the associated worsening morbidity and mortality. It is therefore evident that accurate assessment of severe marrow fibrosis in a BMT specimen (WHO grade MF‐2 or 3) is critical when seeking evidence of sMF or progression of pre‐PMF to overt PMF.^2^

An ongoing challenge in MPN remains stratifying patients into actionable groups that capture our growing understanding of the pathogenic mechanisms underlying disease. To be meaningful, such stratification should enable estimation of the risk of fibrotic or leukaemic progression/transformation. In response, several prognostic scoring systems have been developed, with more recent models incorporating a number of (cyto)genetic parameters in addition to conventional clinical parameters such as thrombotic episodes, deranged blood counts and constitutional symptoms.^7^ The inclusion of comprehensive genetic information has also been used to develop a personalised risk tool for MPN patients that predicts fibrotic progression and transformation to AML.^67^ Combining the predictive power of such approaches with effective, well‐tolerated disease‐modifying therapies has the potential to effectively intercept these most feared consequences of MPN.

### Evaluating therapeutic efficacy in clinical trials

Recent insights into the spectrum of mutations encountered in MPN and the resulting perturbations in cell signalling and cell–cell interactions have led to the identification of myriad new therapeutic targets. Novel agents currently under evaluation or recently approved for clinical use include those directed towards *JAK* signalling, the *PI3K/AKT/mTOR* pathway, apoptotic signalling (*BCL‐2/BCL‐xL*; *SMAC*), TP53 ubiquitination via MDM2, epigenetic regulation, telomerase activity, monocyte differentiation and immunomodulatory signalling (reviewed in refs [22, 23]). Some of these agents have the potential to change the natural history of disease, as seen with the evidence of durable molecular remission in a proportion of *JAK2V617F*‐bearing PV patients treated with interferon or *JAK2* inhibition (ruxolitinib).^68^, ^69^, ^70^

While encouraging, challenges remain over how clinical trials can incorporate novel therapeutic strategies so that new agents can be rapidly and rigorously evaluated using outcome measures that are of tangible benefit to patients. Bone marrow fibrosis represents a key marker for disease activity and progression in many MPNs. However, the only known curative approach for MPN developing severe fibrosis (PMF or sMF) remains allogeneic stem cell transplantation.^71^ Although associated with its own increased risk of morbidity and mortality, transplantation demonstrates that fibrosis can be reversed with extensive manipulation of the marrow microenvironment.^72^ Indeed, novel therapies targeting the drivers of disease present an opportunity to reverse marrow fibrosis or prevent progression to sMF/overt PMF. The ML approaches described previously can be trained to help identify early markers of fibrosis reversal through objective quantification and grading of fibrotic foci. At early stages of disease, ML algorithms, especially those trained using sequential marrow datasets, may be used to identify those patients most at risk of disease progression. This predictive capacity, coupled with appropriate disease‐modifying therapies, could be used to intercept MPN at an earlier stage and reduce the morbidity and mortality associated with fibrotic progression.

Issues remain over the optimal selection of patients for clinical trials and how best to monitor their responses and meaningfully compare treatment arms. The European Leukaemia Net (ELN) Consortium and the International Working Group‐Myeloproliferative Neoplasms Research and Treatment (IWG‐MRT) have issued consensus statements on MPN trial design with the purpose of facilitating communication between regulatory agencies, academic investigators and the pharmaceutical industry.^73^ Central to the design and evaluation of MPN trials is the application of rational and consistent measurements of disease response. This is complicated by the diverse clinical and laboratory manifestations of MPN, each of which may be relevant as a dimension of disease response. Reduction or normalisation of blood counts; reduced organomegaly; disappearance of disease‐related symptoms; loss or reduced molecular disease signature; and improvement in bone marrow histology offer overlapping but differing insights into disease response. Attempts to standardise the measurement and inclusion of these features into MPN response criteria were revised and published in 2013 by the ELN and IWG‐MRT.^74^, ^75^ The inclusion of bone marrow morphological evaluation was advanced as a biologically relevant measure of disease modification. However, definitions of histological disease response remained qualitative, with effects on megakaryocyte hyperplasia (ET), marrow cellularity and reticulin fibrosis (PV) subject to the experience of haematopathologists. Central pathology review was recommended and the working group explicitly cited the difficulty of reaching consensus on the histological definition of remission in MF. Indeed, age‐adjusted normocellularity and grade MF‐1 fibrosis were the only marrow histological features retained as part of the definition for complete (CR) and partial response (PR). In MF, symptom (e.g. total symptom score [TSS] and quality‐of‐life‐based measures), along with spleen volume reduction (SVR), have therefore emerged as the main primary end‐points in most clinical trials. Such an approach has therefore focused on symptom relief rather than improvements in progression‐free survival (PFS), overall survival (OS) or modification of the disease course.

With the development of novel therapeutics, additional end‐points have emerged in an attempt to better evaluate drug efficacy in MPN. These include increased use of patient‐reported outcomes (PRO), OS, PFS, mutation allele frequency (MAF), cytokine modulation, event‐free survival (EFS), leukaemia‐free survival (LFS), transfusion independence and reduction in bone marrow fibrosis. However, incorporating these measures into standardised and meaningful definitions of disease response in MPN represents a significant challenge. The impact of novel therapeutics in MF and the need to address shortcomings in existing disease response criteria have recently been reviewed.^24^ The authors emphasised the lack of standardisation across recent (and ongoing) MF clinical trials evaluating novel agents and highlighted the divergence of disease modification parameters. Moreover, they advanced a refined definition of disease modification that incorporates ‘a clinically meaningful impact on survival outcomes and / or restoration of normal haematopoiesis in conjunction with improvement in bone marrow fibrosis through a substantial and durable reduction in the clonal burden of disease’. The development of AI‐based quantitative assessment of BMT samples is ideally placed to help address this new definition of disease modification in MPN. In turn, it can support and improve the evaluation of new therapies.

## DISCUSSION AND FUTURE PERSPECTIVES

Despite a central role in the diagnosis and assessment of MPN, the assessment of BMT samples by haematologists and haematopathologists remains bound to a set of descriptive and subjective morphological criteria, many of which predate the development of effective treatments. However, novel therapeutics have the potential to modify the course of disease by halting or even reversing the complex cellular and stromal abnormalities that characterise MPN initiation and progression. In order to ensure that only the most effective therapies advance through clinical trials, there is now a clear need to develop rational and reproducible measures of disease response in MPN. Given the clear association between the pathological processes observed in the marrow microenvironment and the clinical course of MPN, it would seem natural to include marrow histological interpretation as a feature of disease modification in future clinical trials. However, incorporation of existing histological criteria relating to marrow cellularity, megakaryocyte hyperplasia/atypia and MF fibrosis grade are subjective and qualitative, with scope for significant interpathologist variation. This makes reliable interpretation of morphological response challenging and risks missing important signals of disease modification, particularly in small‐scale early‐phase trials.

The quantitative bone marrow biopsy analysis outlined in this review has the potential to complement and inform recent insights into the genetic and molecular basis of MPN and may help to address emerging challenges. The identification of numerous mutations in MPN by NGS and whole exome sequencing has raised questions about the significance of certain mutations and their role in determining or modifying the disease phenotype (‘disease drivers’ and ‘clonal drivers’ respectively). Some mutations appear to exert no obvious functional alteration in phenotype (‘passenger’ mutations), with the significance of others remaining uncertain (‘variants of unknown significance [VUS]’), beyond simply providing evidence of clonal haematopoiesis (recently reviewed in ref. [76]). Objectively identifying changes in bone marrow tissue that accompany VUS and comparing them to those of known driver mutations may provide objective evidence of perturbations in bone marrow biology. Such analysis can also inform the experimental functional assays required to unravel the nature of VUS. Improved marrow morphological assessment also has the potential to inform the understanding of ‘clonal haematopoiesis of indeterminate potential’ (CHIP), a potentially premalignant state in individuals with evidence of cancer‐associated mutations but no evidence of a haematological malignancy.^77^, ^78^ The incidence of CHIP depends upon the technical thresholds of mutant allele detection (variant allele frequency [VAF]), but has been estimated at around 3% of the Danish population (VAF >0.01% for *JAK2* *V617F*).^79^ These patients have an ~10‐fold increased risk of developing a haematological malignancy, including MPN, but are also at increased risk of cardiovascular disease (hazard ratio of ~12 for *JAK2 V617F*).^80^, ^81^ Identifying which of these patients are at imminent risk of developing a haematological malignancy and may benefit from early intervention is a significant challenge. However, highly sensitive and accurate approaches to the identification of MPN‐like features in the marrow in patients with CHIP and an objective measure of morphological progression informed by AI approaches could play a significant role. Finally, a comprehensive and quantitative evaluation of the spectrum of marrow morphological features in MPN may help to inform the interpretation of molecular testing at diagnosis and follow‐up. The identification of robust MPN‐like features in patients with a diagnosis of triple‐negative ET may prompt a more exhaustive search for mutations outside of known hotspots in established driver mutations.^67^, ^82^, ^83^, ^84^, ^85^ Conversely, features in keeping with reactive/normal marrow samples may prompt early discharge and/or reduced resource allocation for further molecular testing. Finally, accurately documenting morphological progression in patients with established or advanced disease using serial biopsies will inform our understanding of the genetic prognostic scoring in MPN. The specific morphological correlates of acquired high molecular risk mutations in myelofibrosis (*ASXL1*, *EZH2*, *IDH1*, *IDH2* and *SRSF2*) may be used to help identify and evaluate additional mutations or combinations of mutations also associated with disease progression and impaired survival.^86^

**FIGURE 3:**
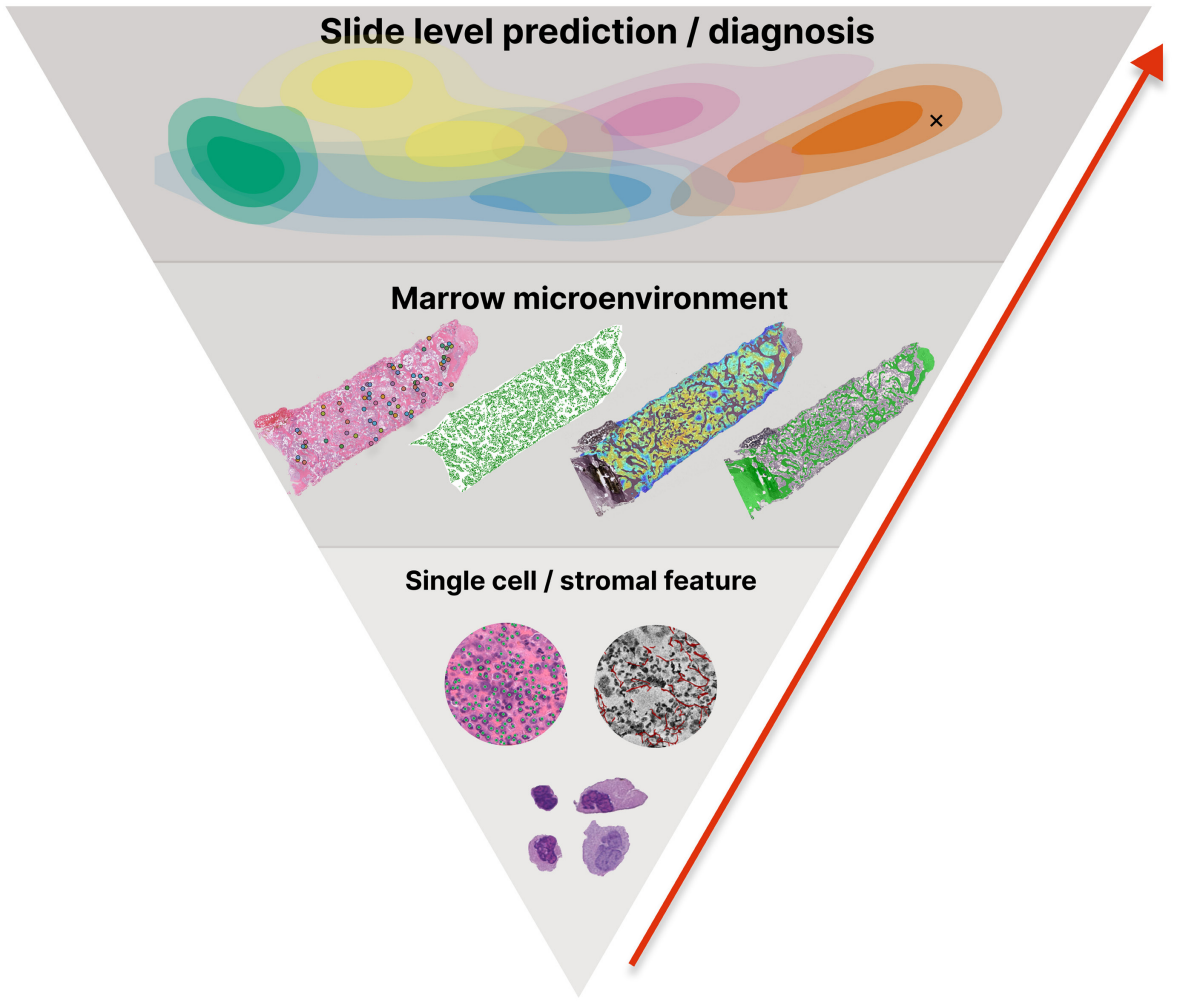
Layered approach to quantitative image analysis of bone marrow trephines in MPNs. Patient samples can be analysed with respect to individual cell or stromal features or represented in terms of biologically relevant tissue microenvironments. For clinical applications, these features can be combined to provide whole slide‐level descriptions that enable individual patient samples (x) to be contextualised against large patient cohorts using computational descriptions of disease space.

The systematic characterisation of cell populations recruited to the marrow microenvironment in MPN and the associated perturbations in cytokine/chemokine signalling can also be explored using biopsy material. The development of spatial transcriptomic approaches employing multiplexed immunofluorescence (IF), next‐generation sequencing (NGS), in situ sequencing (ISS) or in situ hybridisation (ISH) strategies have the potential to transform our understanding of the detailed interactions between the cellular and stromal components of the marrow in health and disease.^87^, ^88^, ^89^, ^90^ While these techniques are currently unsuitable for routine marrow assessment in MPN, they have the power to identify novel features and targets within the marrow that can be translated into future clinical studies. Complementary advances in AI‐driven image analysis of BMTs have the potential to further transform the field with the provision of an objective, quantitative framework for the evaluation of distinct cellular and stromal features that can be combined into an integrated analysis (Figure 3). Sequential patient samples can be directly compared to assess for post‐treatment changes with personalised feature descriptions/measurements contextualised against large patient cohorts.

Notwithstanding the promise offered by AI in the assessment of tissue morphology in MPN, it is sobering to consider that despite numerous publications demonstrating the applications of AI to pathology, only a small number of successful applications for regulatory approval have been made to the US Food and Drug Administration (FDA) or European Medicines Agency (EMA). In part, this can be explained by the fragility of some ML methods when applied to ‘real world’ samples that differ from those used during the initial phases of training and validation. These differences may include technical variation in slide preparation or quality, but can also result from disease variation or heterogeneity that only emerges during the analysis of large and exhaustive datasets.^90^, ^91^ Another potential obstacle is the desire by regulatory agencies to ensure the basic explainability of how AI software works, with a perceived lack of interpretability of deep neural networks. However, these barriers can be overcome with suitably robust algorithm validation, as evidenced by the FDA's recent approval of Paige Prostate for AI‐based histological diagnosis of prostate cancer. The extent to which the bone marrow AI algorithms outlined in this article can follow this pathway of approval remains unclear, as does the likely timescale. Indeed, while determining the accuracy of certain tasks such as the detection of bone marrow fibrosis may be relatively straightforward and require limited validation studies, demonstration of improved MPN diagnostic quality may require more exhaustive prospective clinical trials. Some of these issues have recently been reviewed in the specific context of bone marrow biopsy evaluation.^55^ More generally, as growing numbers of AI‐based pathology reporting algorithms proceed to regulatory approval, it can be expected that guidelines and recommendations will emerge that support translational pathology groups. Such stage‐specific reporting guidelines for the early and live clinical evaluation of AI have already emerged in the comparatively mature field of AI‐driven radiology (reviewed in ref. [92]).

## AUTHOR CONTRIBUTIONS

Daniel Royston drafted the paper and Hosuk Ryou, Oliver Lomas, Helen Theissen, Emily Thomas and Jens Rittscher critically appraised and revised its contents. Figures were generated by Hosuk Ryou, Helen Theissen and Emily Thomas.

## FUNDING INFORMATION

J.R. is supported through the EPSRC‐funded Seebibyte programme (EP/M013774/1) and is an adjunct professor of the Ludwig Institute for Cancer Research, Oxford Branch. D.R. is supported by Cancer Research UK and Blood Cancer UK; H.R. is supported by the CRUK Oxford Centre. E.T. is funded by the EPSRC Centre for Doctoral Training in Sustainable Approaches to Biomedical Science.

## CONFLICT OF INTEREST STATEMENT

J.R. is a cofounder and equity holder of Ground Truth Labs Ltd. D.R. provides consultancy services to Ground Truth Labs Ltd. and Johnson & Johnson. The remaining authors declare no competing interests. The funders had no role in the conception or writing of this manuscript.
